# Assessing Health Risks Associated with Heavy Metals in Food: A Bibliometric Analysis

**DOI:** 10.3390/foods12213974

**Published:** 2023-10-30

**Authors:** Elena L. Ungureanu, Andreea L. Mocanu, Corina A. Stroe, Denisa E. Duță, Gabriel Mustățea

**Affiliations:** National Research & Development Institute for Food Bioresources, 020323 Bucharest, Romania; elena_ungureanu93@yahoo.com (E.L.U.); andreea.mocanu1@yahoo.com (A.L.M.); corina10_2010@yahoo.com (C.A.S.); denisa.duta@bioresurse.ro (D.E.D.)

**Keywords:** bibliometrics, food products, health risk assessment, heavy metals, VOSviewer

## Abstract

Bibliometric analysis is an effective method used to identify research trends based on historical publications that involves combining different frameworks, tools and methods, leading to the creation of different metrics. This study employed bibliometric analysis to investigate the global health risk assessment of heavy metals in food from 2000 to 2022 using Web of Science and VOSviewer. We explore publication trends, affiliations, countries, journals, citations, keywords and author collaborations. Of the 573 publications on this topic, there has been a notable increase in recent years. The Ministry of Agriculture and Rural Affairs (China) and Shahid Beheshti University of Medical Sciences (Iran) are the most prolific affiliations. *Environmental Science and Pollution Research* is the top journal. Notably, “heavy metals”, “risk assessment”, “cadmium”, “lead”, and “trace elements” are frequently used keywords. A study by Miraglia et al. in 2009 received the most citations. Amin Mousavi Khaneghah (Poland) is the most prolific author, with 24 papers. Articles mainly focus on contamination levels in fish, seafood, cereals, dairy, meat, and fruit/vegetables. Some studies highlight potential risks, necessitating stricter food product controls for consumer safety.

## 1. Introduction

Food serves as a primary source of essential nutrients for the body, but it can also contain some nonnutritive substances that are not only unnecessary but potentially harmful to human health. Besides the various minerals found in our daily diet, toxic heavy metals such as Cd, Pb, Hg, and Ni can be found in food products. These elements can be toxic, even at relatively low concentrations [1]. The issue of heavy metal contamination in the environment and the food chain is widespread. These chemical contaminants can be absorbed by plants, potentially entering the food chain and exposing humans to them. Based on their toxicity to living organisms, heavy metals can be ranked in the following order: Hg > Cu > Zn > Ni > Pb > Cd > Cr > Sn > Fe > Mn > Al [2]. According to Sarsembayeva et al., 2020 [3], the level of contaminants in food raw materials has increased almost fivefold in the last 5 years, with toxic elements being found in 90% of commonly studied food products. Consequently, there is a need to deepen our understanding of the ways in which food is contaminated with heavy metals and to improve processing techniques aimed at mitigating the adverse effects of food contaminants.

In recent decades, significant research has been conducted on heavy metal emissions, pollution, health risk assessments and mitigation strategies. It is crucial to have a comprehensive understanding of the risks of heavy metals to human health, as well as to identify emerging research trends, to monitor key research areas, and to outline future research directions in this field [4].

Bibliometric analysis is an effective method used to identify research trends based on historical publications, and has been used in many fields of science and engineering. However, few studies have focused on the health risks of heavy metals based on bibliometric analysis, in particular bibliometric studies on heavy metals in food products [4]. This analysis involves the integration of various frameworks, tools, and methods for studying and analyzing citations in publications, resulting in the development of various metrics. These metrics are used to understand the intellectual landscape of different academic fields and evaluate the impact of scientific journals, research studies, and researchers [5].

The aim of this study was to use bibliometric analysis and network visualization to provide an overview of the current state of research in the field of health risk assessment of heavy metals in food. The analysis was performed using publications from the Web of Science (WoS) Core Collection database. The second section presents the data sources and research methods used. The third section presents a comprehensive discussion of the research results, including annual publication distribution, author contributions and collaborations, influential institutions, most productive countries, and keyword co-occurrence analysis, and the fifth section presents the relevant conclusions.

## 2. Data Collection and Search Strategy

In this study, bibliometric analysis was utilized for data extraction, as bibliometrics is a method that combines mathematics, statistics, and related disciplines to analyze documents and uncover relationships among them [6]. Article details, such as affiliations, countries, authors, journals and research area, and keywords, were extracted from WoS.

The research was carried out in September 2023 using the following keywords: “health risk assessment” (all fields), “heavy metals” (all fields), “food products” (all fields), and the publication interval “2000–2022”. These keywords were used to search for relevant papers, and the search included paper titles, abstracts, keywords, affiliations, authors, sources, and cited references. A total of 573 articles that met the selection criteria were chosen for the analysis.

Co-authorship networks of authors, countries, institutions, keyword, co-occurrence networks of all keywords, author keywords and keywords, citations of documents, authors, sources, organizations, and countries were analyzed using VOSviewer (version 1.6.19) software, a free mapping program used for conducting bibliometric analysis.

## 3. Results

### 3.1. Trends in Global Publications

Our evaluation of human health risks entailed an assessment of the potential adverse health effects in individuals exposed to heavy metals through various pathways. This process involved assessment of daily exposure and the evaluation of both noncarcinogenic and carcinogenic risks [7]. Many researchers in the literature [8,9,10,11,12,13,14,15] have used this method to estimate the potential adverse health effects resulting from exposure to contaminated products [16].

Analyzing the number of papers published within a specific period is a valuable approach for assessing the trends and extent of development in a particular field [17].

**Figure 1:**
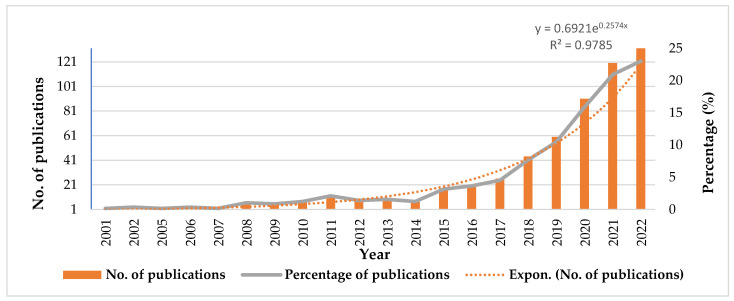
Dynamics of publications on health risk assessment of heavy metals from food products between 2000 and 2022.

The publication statistics for the identified papers are presented in Figure 1. When analyzing the publication trends over time (from 2000 to 2022), we can approximate four distinct stages according to the model of Zhang et al., 2022 [17]. In stage one (2000–2014), with the exception of 2011, there were fewer than 10 papers published annually. This phase started from the first article published by Lee et al., 2001 [18], until 2014, and only a limited number of papers (61) were published, constituting only 10.65% of the total publications during this period. In stage two (2015–2018), there was a gradual increase in the number of papers published per year, ranging from fewer than 10 to almost 50 papers annually. Between 2015 and 2018, there was a notable increase in publications: from 18 papers (3.14%) in 2015 to 44 papers (7.68%) in 2018. In stage three (2019–2020), there was a sustained gradual increase, with an annual publication count ranging from over 50 papers to just under 100 papers. The number of publications varied between 60 papers (10.47% in 2019) to 91 papers (15.88% in 2020). In the last stage, between 2021 and 2022, the number of articles published each year exceeded 100 papers, ranging from 120 papers (20.94% in 2021) to 132 papers (23.04% in 2022). Notably, the publications related to the health risk assessment of heavy metals from food products increased significantly, from just 1 paper in 2001 to 132 papers in 2022. The top three years in terms of published papers were 2020 (91, 15.88%), 2021 (120, 20.94%), and 2022 (132, 23.04%). As can be seen from Figure 1, in 2000, no article was published. This trend analysis highlights the growing concern over the past two decades regarding the adverse health effects of heavy metal ingestion through contaminated food products.

The majority of publications (475, 82.89%) were scientific articles, followed by review articles (88, 15.36%) of the total publications in the field, early access articles, proceedings, editorial material, and lastly book chapters (2, 0.35%). Notably, English was the predominant language, representing 99.47% of all publications, while the remaining 0.63% of papers were published in Chinese.

Regarding WoS categories, the majority of papers were in the environmental science field (264, 46.07%), followed by fields such as food science technology (149, 26.0%), toxicology (89, 15.53%), applied chemistry (11.17%), and environmental engineering (33, 5.76%). This distribution highlights that studies were focused on environmental concerns, toxicology, and the quality of food products.

Table 1 provides an overview of the bibliographic statistics for these studies. The data reveal that the average number of co-authors per article is 5.39, which highlights the collaborative research in this field. On average, each article receives 23.08 citations, signifying varying levels of citation rates across the articles. The dataset also includes 3089 authors of single-authored articles. Additionally, there are 246 authors appearing on 2 papers, 71 on 3 papers, 29 on 4 papers, 17 on 5 papers, 11 on 6 papers, 10 on 7 papers, 5 on 8 papers, and 4 on 9 papers. Out of the 573 papers, 558 were the work of multiple authors, while only 15 papers were authored by a single author. It should be mentioned that the average number of authors per paper can vary significantly depending on the scientific field or discipline [19].

The majority of risk assessment studies have been conducted on total diet [21,22,23,24], ready-to-eat products [25], fish and seafood products [26,27,28], fruits and vegetables [29,30,31,32], cereals and cereal products [33,34,35], dairy and dairy-derived products [36,37,38], as well as meat and meat products [39,40,41]. Also, studies have been conducted on risk assessment of heavy metals from eggs [42,43,44], cocoa and cocoa products [45,46], sesame [47], pistachio [48], peanut seeds [49], spices [50], salt [51], chewing gum, peppermints, and sweets [52], honey [53], water and drinks [54,55,56], food supplements [57], tea products [58] and infant formula [59].

The hazard quotient (HQ), target total hazard quotient (TTHQ) and hazard index (HI) were higher than limit 1 in many research articles. An HQ and HI higher than 1.0 from one or more exposure routes is considered unacceptable, indicating that the exposed population may be exposed to noncarcinogenic effects and risk mitigation measures should be implemented [60]. In the case of adults, the studies demonstrated that the women were more susceptible to noncarcinogenic risk compared to men, and children more sensible than adults (HQ > 1, HI > 1).

For cancer risk (CR) and total cancer risk (TCR) many of articles demonstrated that the levels of heavy metals found in tested products were found in the unacceptable level. Also, the children were more susceptible to carcinogenic risk associated with consumption of tested products compared with adults. Pipoyan et al., 2019 [61] classified cancer risk into four groups: high risk (≥0.001), moderate risk (≥0.0001 but <0.001), low risk (≥0.000001 but <0.0001) and very low risk (<0.000001). The carcinogenicity of tested products was produced by the levels of As, Cd or Pb.

### 3.2. Distribution of Institutions

Scientific collaboration can be described as the interaction occurring in a social context among two or more scientists, which facilitates the exchange of ideas and the achievement of common objectives related to a shared goal. Collaboration among scientists is motivated by the chance to discover new knowledge, the growing specialization within scientific fields, the need to develop infrastructure, and the necessity to combine diverse knowledge and skills. Such collaboration can expand the horizons of a research project and promote innovation by granting access to various disciplines. Co-authorship analysis remains a widely used method to understand and evaluate patterns of scientific collaboration. This form of analysis enables the evaluation of research program productivity, the examination of the connection between scientific and technological advancement, the mapping of priority thematic areas, the assessment of regional contributions to knowledge generation, the evaluation of inter-organizational networks, and evaluation of international collaboration [62].

Within co-authorship networks, nodes represent authors, organizations, or countries, and connections are established when they contribute to the authorship of a paper. Collaborative networks are very important in health innovation due to their complexity, which includes the participation of numerous stakeholders and an increasing dependence on interdisciplinary research. This analysis allowed the identification of leading academic researchers in the field and research organizations that have played an important role in disseminating information and the allocation of resources for health management [54]. Co-authorship networks serve as valuable bibliometric indicators for examining various patterns of collaboration within academic disciplines. These networks can be analyzed using various measures of social network analysis (SNA) [63].

The distribution of institutions that contributed to publications in this field was analyzed using VOSviewer software. Out of a total of 1131 organizations that have contributed to 573 articles, 262 authors from organizations in library and information science meet the criteria of contributing two or more articles in collaboration with other organizations.

Among institutions, the Ministry of Agriculture and Rural Affairs had the highest number of publications, accounting for 32 papers (5.58% of the total). This was followed by Shahid Beheshti University of Medical Sciences with 27 papers (4.71%), the Chinese Academy of Sciences with 25 papers (4.36%), the Egyptian Knowledge Bank EKB with 21 papers (3.66%), and Tehran University of Medical Sciences with 19 papers (3.32%), while other institutions contributed less than 3% of the publications.

**Figure 2:**
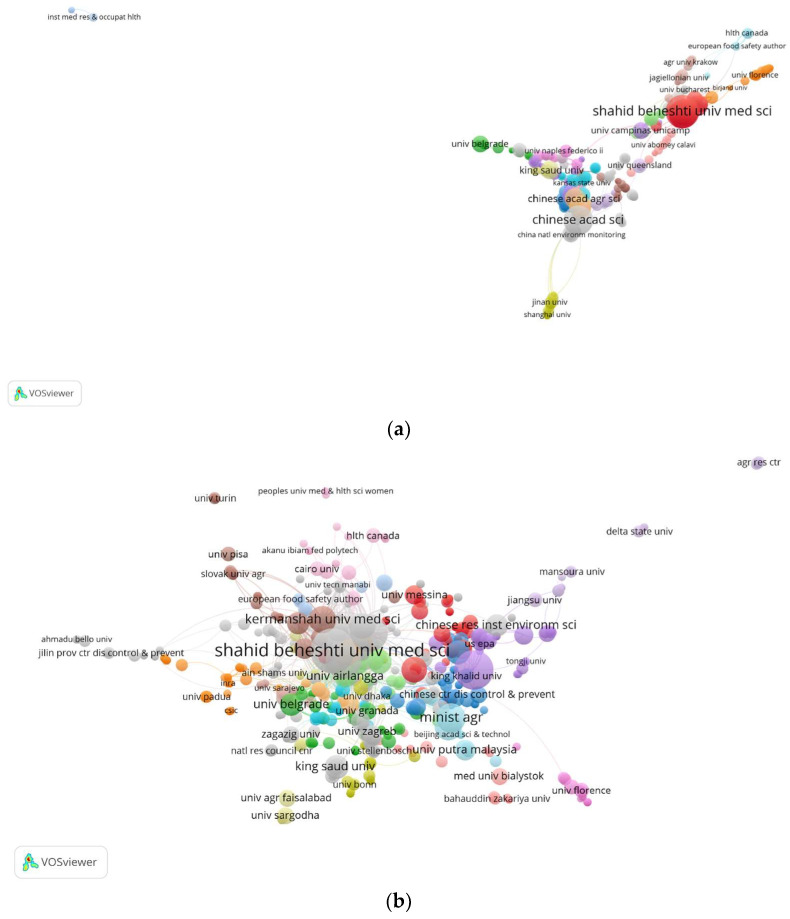
Analysis of affiliations. (**a**) The collaborations between organizations. (**b**) Organizations’ citation.

The co-authorship organizations were analyzed using VOSviewer software, resulting in the network visualization presented in Figure 2a. In this visualization, a total of 585 items were categorized into 32 clusters, representing nodes and links based on their strength. The thickness of the links depends on the number of articles published through co-authorship collaborations [64]. Notably, the Ministry of Agricultural Rural Affairs, Shahid Beheshti University of Medical Sciences, Chinese Academy of Sciences, and Egyptian Knowledge Bank (EKB) emerged as the top organizations in terms of articles published with co-authorship in the field of health risk assessment of heavy metals in food products. They were followed by Tehran University of Medical Sciences, Hormozgan University of Medical Sciences, Universidade Estadual de Campinas, Chinese Academy of Agricultural Sciences, and Kermanshah University of Medical Sciences, among many others. Among the total of 573 articles, 100 publications were authored by individuals who were affiliated with the same institution.

A total of 182 papers were authored by researchers from different organizations. The initial article published by authors from various organizations but the same country was the research conducted by Chang et al., 2005. This study focused on examining contaminants in fresh fruits and vegetables from Taiwan and introduced the hazard analysis critical control point (HACCP) system to agricultural product industries and growers to enhance the safety of their produce.

WoS data indicate a recent increase in inter-organizational collaborations. The benefits and risks of such collaborations are derived from various theories, including transaction costs economics, the resource-based view, social network theory, and organizational learning. These theories offer different perspectives on inter-organizational cooperation, allowing us to compile a list of its advantages and disadvantages. All four approaches suggest a positive connection between inter-organizational cooperation and organizational innovation [65].

In Figure 2b, the citation network of organizations is presented. VOSviewer software detected 53 clusters and 781 organizations that cited the affiliations from the study. Shahid Beheshti University of Medical Sciences received the highest number of citations (861), followed by Chinese Academy of Sciences (633), University of Granada (559), Kermanshah University of Medical Sciences (489), Tehran University of Medical Sciences (452), and Semnan University of Medical Sciences (408). The remaining affiliations received fewer than 400 citations. Cluster 1, consisting of 53 organizations, had the highest number of documents (97) citing the organizations from the bibliometric study.

### 3.3. Country Collaboration Analysis

Country co-authorship analysis is a valuable method for understanding collaboration between countries and identifying influential contributors in a specific field [17].

The data obtained from WoS and VOSviewer indicate that China had the highest number of published documents in this field, with 149 papers (26% of the total). This was followed by Iran with 67 papers (11.695%), Italy with 50 papers (8.70%), and the USA with 47 papers (8.20%). It is notable that South Korea, despite not being among the most productive countries in this field, published its first article on this topic in 2001 [18]. Among the most productive countries (Table 2), Spain and Germany published their first articles on this topic in 2002, followed by Italy in 2006, the USA in 2008, Poland in 2009, China in 2010, India in 2011, Brazil in 2012, Iran in 2016, and Pakistan in 2017.

China, despite its late start in 2010 with the publication of Liu et al., 2010 [66], has become the most prolific country in this field. Between 2010 and 2022, China published 149 articles on the health risk assessment of heavy metals in cereal products, constituting nearly one-quarter of all publications on this subject (as shown in Table 2). A significant increase in the number of articles published by China in this area was between 2000 and 2022, with 80 articles, which accounts for 53.69% of the total papers of this country.

An increasing number of research papers indicate that China can lead in research on the risk of heavy metals, encompassing both academic investigations and practical field applications. This trend can be attributed to several factors. Firstly, residents from developing countries are becoming more conscious about environmental and health issues due to the pollution that results from the rapid industrial expansion over recent decades. Secondly, in developed countries, improved technologies and the relocation of production to other countries have resulted in less severe heavy metal pollution compared to the situation in developing countries [6].

Table 2 indicates that the USA received the highest number of citations, with an average citation rate of 41.64, followed in descending order by Germany (38.08), Spain (34.70), and Italy (32.47). In contrast, Poland had the lowest average citation rate for its published papers, at 16.44.

**Table 2:** Top 10 productive countries on research associated with health risk assessment of heavy metals from cereal products, based on authors’ affiliation.

Rank	Country	Articles	Percent (%) *	Citations	Citation per Document	H-Index_2022_ ** of Countries
1	China	149	26.0	4128	27.70	1210
2	Iran	67	11.69	1647	24.58	445
3	Italy	50	8.73	1623	32.46	1255
4	USA	47	8.20	1957	41.64	2880
5	Poland	39	6.81	641	16.44	687
6	Brazil	35	6.11	731	20.89	729
7	Spain	33	5.76	1145	34.70	1127
8	Germany	26	4.54	990	38.08	1584
9	India	26	4.54	752	28.92	795
10	Pakistan	23	4.01	599	26.04	381

*—Percentage from the total number of publications (*n* = 573); **—https://www.scimagojr.com/countryrank.php?year=2022 (accessed on 25 August 2023) [67].

**Figure 3:**
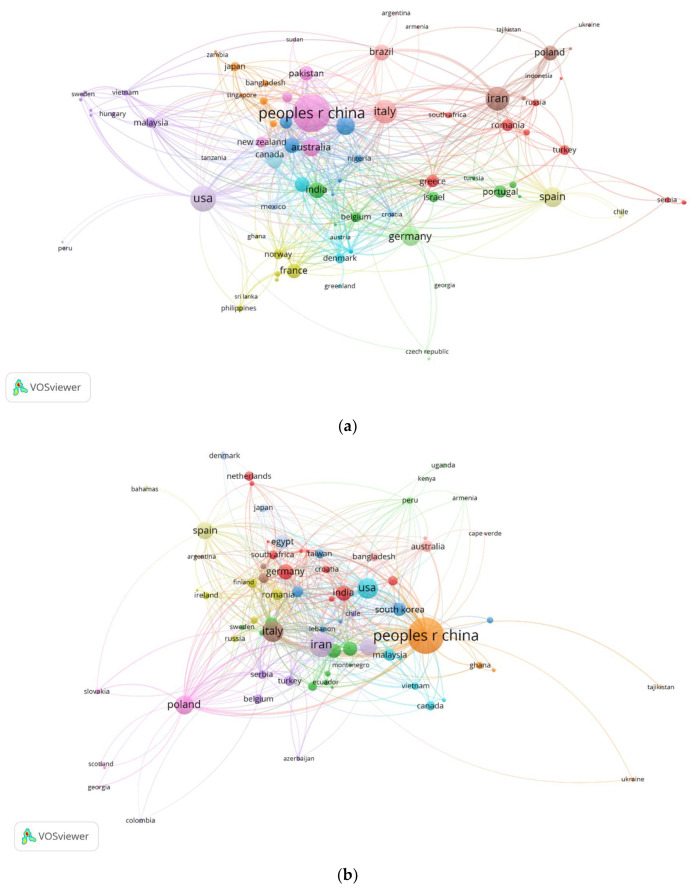
Country analysis. (**a**) International collaborations of countries. (**b**) Country citations.

Figure 3a illustrates the distribution of publications and collaborative relationship among countries involved in the study of health risk assessment related to heavy metals in food products from 2000 to 2022. Each country is represented by a distinct circle, the circle size indicating the number of articles published. The presence of connections between countries signifies the frequency of collaboration between them, and the length of the connecting lines reflects the proximity of this collaborative relationship [17].

The WoS analysis indicated that 282 papers (49.21%) were elaborated by researchers from a single country. The first article in this category was the study by Lee et al., 2001 [10]. This article examined various aspects related to chemical residues in food products, including their sources, adverse effects, chemical residue status in different countries, toxicological and pharmacokinetic backgrounds of maximum residue limits (MRLs), as well as nongovernmental efforts to reduce chemical residues in food.

The remaining 291 papers (50.78%) were the result of international collaboration. The first article published by authors from different countries was the study conducted by Pesch et al., 2002 [68], which investigated the risk of arsenic exposure on humans based on residential history, annual emissions, nutritional habits, and arsenic content in food in the district of Prievidza, Slovakia.

According to Han et al., 2020 [4], there has been a significant increase in international cooperation in the field of heavy metal health risks in recent years, most probably due to the increasing concern on environmental contamination and public health risks associated with heavy metals, as well as the need for actions to combat these problems.

A total of 95 countries conducted research in this field, resulting in 15 clusters and 533 links between these countries. These clusters indicate numerous dispersed cooperative relationships among these countries. The strength of international collaboration can be reflected by the number of links. According to the data from VOSviewer, the USA has the highest number of links with other countries (48), followed by Italy (44 links), England (38 links), Germany (38 links), Spain (37 links), India (35 links), Egypt (32 links), China (31 links), South Korea (28 links), Belgium (27 links), Malaysia and Denmark (24 links), Greece and Nigeria (23 links), Iran (22 links), Brazil (20 links), and Pakistan (18 links). Notably, although Brazil, Pakistan, China, and Iran are among the top ten most productive countries in terms of the number of publications, they have not engaged in extensive international collaboration.

Figure 3b displays the citation network of countries. The VOSviewer software identified 14 clusters and 495 countries that cited the countries included in the study. The People’s Republic of China received the highest number of citations (4128 citations), followed by the USA with 1957 citations, Iran with 1647 citations, Italy with 1623 citations, Spain with 1145 citations, Germany with 990 citations, South Korea with 961 citations, and Australia with 956 citations. The remaining countries received fewer than 900 citations. Interestingly, South Korea and Australia, despite not being among the top 10 most prolific countries, are among the 8 most cited countries.

### 3.4. Analysis of Journals and Research Areas

The analysis examined the trends in journal preferences from 2000 to 2022 by considering the number of publications related to health risk assessment of heavy metals in food products.

A total of 573 articles on the health risk assessment of heavy metals from food products were published across 189 journals. Table 3 presents information about the 10 most productive journals in this field, including their H-index, impact factor, quartile, and Journal Citation Reports partition. *Environmental Science and Pollution Research* had the highest number of articles (36 papers, 6.28%), having an H-index2022 of 154. It was followed by *Science of the Total Environment* (25 papers, 4.36%), which had an H-index2022 of 317, *Journal of Food Composition and Analysis* (23 papers, 4.01%) with an H-index2022 of 130 and the *International Journal of Environmental Research and Public Health* (22 papers, 3.84%) with an H-index2022 of 167. As regards the citation means, *Food and Chemical Toxicology* obtained the highest mean citations per document, 66.47, followed by *Science of the Total Environment*, with 32.2 citations per document, *Food Additives and Contaminants, Part A: Chemistry, Analysis, Control, Exposure, and Risk Assessment*, with 29.47 citations per document. On the other hand, articles published in *Environmental Geochemistry and Health* had the lowest citation mean of 7.57 (Table 3).

In the context of journals, approximately 31 journals published a minimum of five papers, contributing to over half of the total publications (350 papers, 61.08%). Additionally, as indicated in Table 3, the papers published in the 10 most prolific journals accounted for 36.13% (207 papers) of the overall publications. These findings highlight the extensive interest and involvement in research related to environmental and food safety [69].

**Figure 4:**
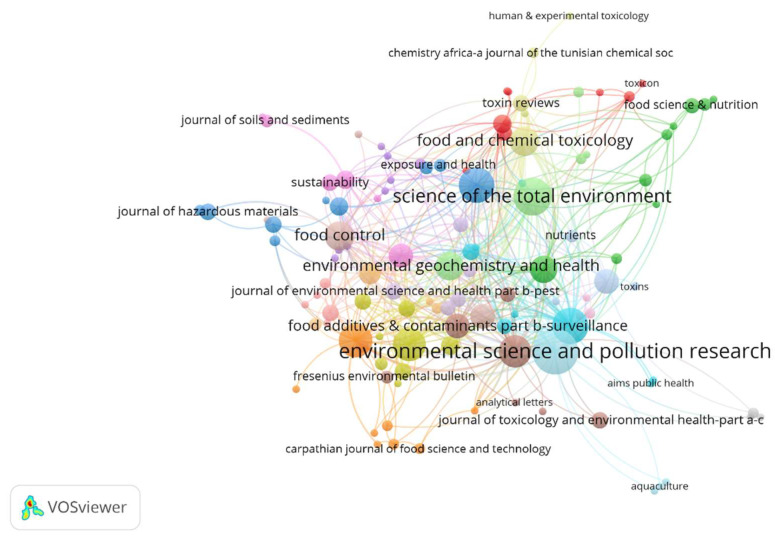
Analysis of journals—network visualization of the most cited journals.

The citation network of countries is illustrated in Figure 4. The VOSviewer software identified 19 clusters and 407 journals that cited the journals included in the study. The *Food and Chemical Toxicology Journal* received the highest number of citations, with 997 citations, followed by *Science of the Total Environment* with 805 citations, *Environmental Science and Pollution Research* with 715 citations, *Environment International* with 610 citations, *Ecotoxicology and Environmental Safety* with 590 citations, and *Food Additives and Contaminants, Part A: Chemistry, Analysis, Control, Exposure, and Risk Assessment*, with 560 citations. The remaining journals received fewer than 500 citations. Interestingly, *Environment International* and *Ecotoxicology and Environmental Safety*, despite not being among the top 10 journals in terms of the number of papers published, are among the 6 most cited journals.

### 3.5. Analysis of Publications

In bibliometric research, citation analysis is widely used as an indicator for assessing the quality of papers, representing the impact of a study and the level of attention it attracts in academic circles [6].

**Figure 5:**
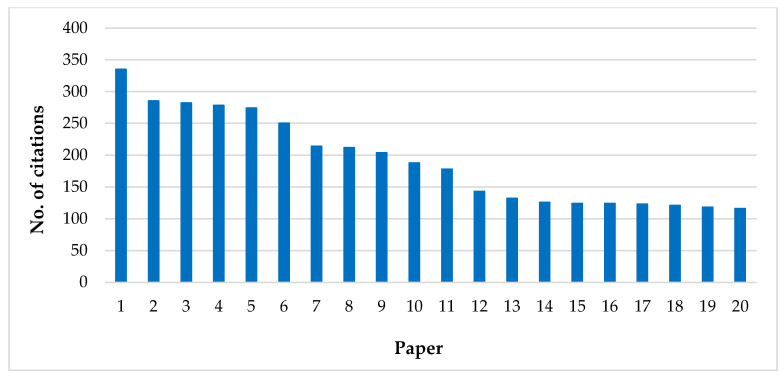
Top 20 cited publications in the field of health risk assessment of heavy metals from food products. 1—Miraglia et al., 2009 [70], 2—Hussain et al., 2017 [71], 3—Lu et al., 2017 [72], 4—Olmedo et al., 2013 [73], 5—Kumar et al., 2020 [74], 6—Yu et al., 2012 [75], 7—Rajeshkumar and Li, 2018 [76], 8—Zwolak et al., 2019 [2], 9—Awasthi et al., 2016 [77], 10—Rose et al., 2010 [78], 11—Mao et al., 2019 [79], 12—Thompson and Darwish, 2019 [80], 13—Antoniadis et al., 2019 [81], 14—Fathabad et al., 2018 [82], 15—Zhang et al., 2018 [83], 16—Tsatsakis et al., 2017 [84], 17—Wiesinger et al., 2021 [85], 18—Olmedo et al., 2013 [86], 19—Grant and Sheppard, 2008 [87], 20—Langie et al., 2015 [88].

This analysis is exemplified in the top 20 cited documents from 2000–2022, presented in Figure 5. Ranked first with 335 citations is the article by Miraglia et al., 2009 [70], elaborated by researchers from Italy, the Netherlands, and Germany, titled “Climate change and food safety: An emerging issue with a special focus on Europe”. This review article presents the food safety concerns that are influenced by climate change, particularly in Europe. It discusses various issues, including mycotoxins formed on plant products in the field or during storage, residues of pesticides in plant products affected by changes in pest pressure, trace elements and heavy metals in plant products depending on changes in their abundance and availability in soils, polycyclic aromatic hydrocarbons in foods due to changes in long-range atmospheric transport and deposition into the environment, marine biotoxins in seafood resulting from the production of phycotoxins by harmful algal blooms, and the presence of pathogenic bacteria in foods due to more frequent extreme weather conditions.

This most highly cited publications were followed, in decreasing order, by Hussain et al., 2017 [71] (with 286 citations), Lu et al., 2017 [72] (with 282 citations), Olmedo et al., 2013 [73] (with 278 citations), Kumar et al., 2020 [74] (with 274 citations), Yu et al., 2012 [75] (with 250 citations), Rajeshkumar and Li, 2018 [76] (with 214 citations), Zwolak et al., 2019 [2] (with 212 citations), and Awasthi et al., 2016 [77] (with 204 citations). The remaining papers received fewer than 200 citations per document.

Is important to highlight that among the 20 most frequently cited publications, 13 were original articles. These papers were focused on the distribution and accumulation of various heavy metals in different products, including fish and shellfish samples, aquatic organisms across different trophic levels, total diet, rice, maize, processed fruits, and diverse geographical areas such as the Canary Islands, Taihu Lake in China, the UK, the Yangtze River Delta, Greece, Tehran, and the risk assessment associated with these contaminants for different categories of consumers. This aspect highlights the growing interest among researchers in understanding the human health risks associated with heavy metals in food products and their sources of contamination.

Additionally, the proportion of review articles in this context, which accounts for 35% (seven papers), is negligible. According to Gao et al., 2022 [6], the rising percentage of review articles suggests the presence of authoritative and comprehensive evaluations of concepts, characteristics, and influencing factors within this field. These reviews are expected to provide a solid groundwork for future research endeavors.

**Figure 6:**
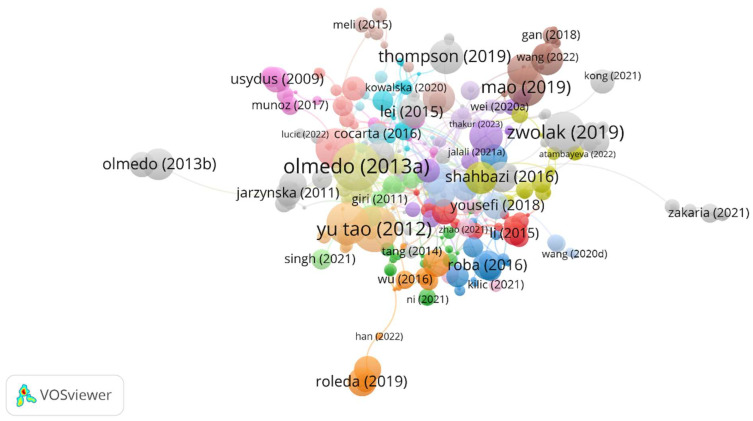
Network visualization of documents citation. (Cluster 1: Li (2015) [89]; Zhao (2021) [43], Cluster 2: Ni (2021) [90]; Cluster 3: Roba (2016) [91], Cluster 4: Atambayeva (2022) [92], Cluster 5: Jalali (2021) [93], Cluster 6: Kowalska (2020) [94], Cluster 7: Roleda (2019) [95]; Han (2022) [96]; Wu (2016) [97]; Cluster 8: Gan (2018) [98], Wang (2022) [99], Cluster 9: Usydus (2009) [100], Munoz (2015) [22], Cluster 11: Singh (2021) [101], Giri (2011) [102], Cluster 12: Yousefi (2018) [103], Wang (2020) [104], Cluster 13: Olmedo (2013) [73], Cluster 14: Wei (2020) [105], Cluster 15: Cocarta (2016) [106], Cluster 16: Yu Tao (2012) [75], Cluster 17: Meli (2015) [107]; Cluster 18: Kilic (2021) [108], Cluster 19: Shahbazi (2016) [109], Cluster 20: Lei (2015) [110], Lucic (2022) [31], Cluster 21: Jarzynska (2011) [111], Cluster 22: Zwolak (2019) [2], Cluster 23: Zakaria (2021) [112], Cluster 24: Kong (2021) [113], Cluster 25: Tang (2014) [114], Cluster 26: Thompson (2019) [80], Cluster 27: Olmedo (2013) [86].

The citation network of the papers is presented in Figure 6. VOSviewer software revealed 28 clusters and 366 items that cited the documents from the study.

### 3.6. Analysis of Authors

Analyzing co-authors can be a valuable tool for understanding the structure of academic networks. These co-authors often come from diverse backgrounds and regions, collaborating to advance scientific knowledge in their respective fields. Such scientific cooperation contributes to the generation of new knowledge. The analysis of co-authors serves as a crucial indicator of collaboration and is an essential component of bibliometric analysis [115]. In this section, we used the VOSviewer software to analyze co-authorship patterns, providing insights into the collaborative relationships among authors.

The most productive authors were as follows: Amin Mousavi Khaneghah from Poland (24 papers, H-index 54), Yadollah Fakri from Iran (19 papers, H-index 43), Nabi Shariatifar from Iran (9 papers, H-index 29), Ndiye Kebonye from Germany (8 papers, H-index 10), Prince Chapman Agyeman from the Czech Republic (8 papers, H-index 9), Kingsley John from Canada (8 papers, H-index 11), Lubos Boruvka from the Czech Republic (8 papers, H-index 8), Radim Vasat from the Czech Republic (7 papers, H-index 15), Hedayat Hosseini from Iran (7 papers, H-index 36), and Trias Mahmudiono from Indonesia (7 papers, H-index 9).

**Figure 7:**
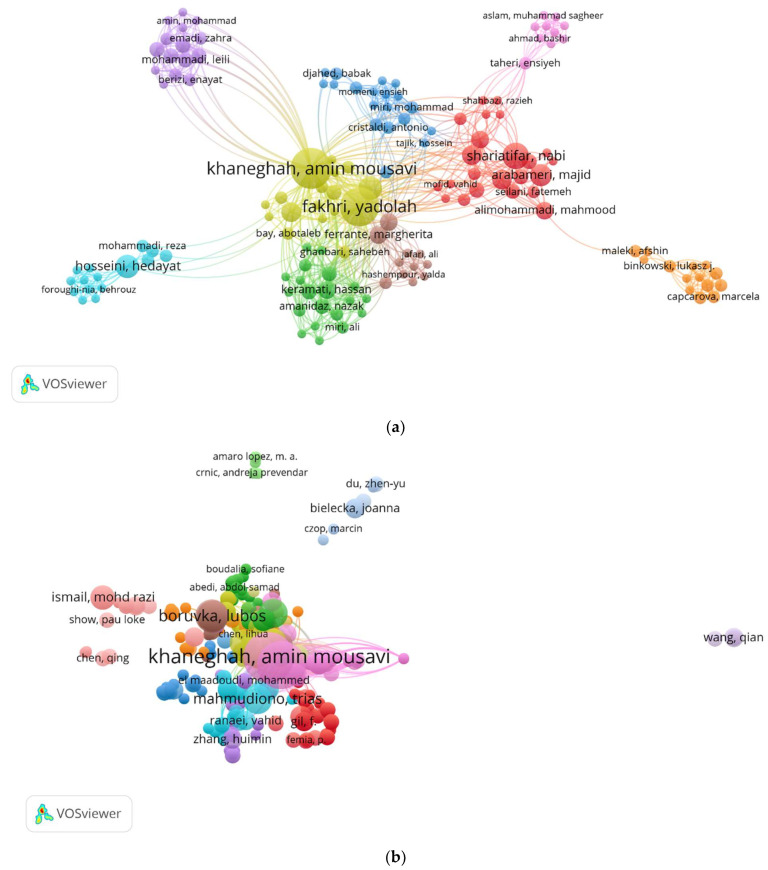
Authors analysis. (**a**) Co-authorship network visualization between the most connected authors. (**b**) Author citations.

The co-authorship analysis, as depicted in Figure 7a, revealed a total of 19 clusters, 1325 links and an overall link strength of 1530. Notably, each cluster is represented by a prominent author:

- The first cluster (red) is associated with Shariatifar Nabi.
- The second cluster (green) is linked to Ferrante Marhjerita.
- The third cluster (blue) is represented by Hosseini Hedayat.
- The fourth cluster (yellow) is tied to Keramati Hassan.
- The fifth cluster (purple) is connected to Fathabad Ayub Ebadi.

Cluster 6 (turquoise) includes the two most prolific authors, Amin Mousavi Khaneghah and Yadollah Fakri. This analysis provides valuable insights into the collaborative patterns and leading authors within the field.

The citation network of authors is depicted in Figure 7b. Using VOSviewer software, 14 clusters and 14,032 links citing the authors from the study were identified. Yadolah Fakhri from Iran received the most citations, 674, followed by Amin Mousavwi Khaneghah from Poland with 566 citations, Hernandez Antonio F. from Spain with 559 citations, and Pla Antonio, Olmedo Pablo, and Gil Fernando, all from Spain, each with 435 citations. Other authors received fewer than 400 citations. Notably, Hernandez Antonio F, Pla Antonio, Olmedo Pablo, and Gil Fernando, despite not being among the 10 most prolific authors in this field in terms of the number of papers published, are among the top 6 most cited authors.

### 3.7. Analysis of Keywords

Keywords serve as theme indicators for published articles, and conducting bibliometric analysis on high-frequency keywords helps identify research hotspots and provides insights into the evolutionary context within a specific field [6].

Keyword co-occurrence analysis is a bibliometric method used to identify associations between topics within a particular research field. This tool helps in uncovering relationships and connections between various items or pairs, and it quantifies the strength of these connections. A higher numerical value of a link indicates a stronger connection between items or topics [116].

VOSviewer records contain three categories of keywords: All Keywords, Author Keywords and Keywords Plus. Author Keywords consist of a selection of terms chosen by the authors to best describe the content of their paper, while Keywords Plus are words or phrases that are extracted from the titles of the references cited in the article using an automated computer algorithm developed by Thomson Reuters, even if they are not present in the title of paper or listed as Author Keywords. Analyzing both Keywords Plus and Author Keywords when examining the knowledge structure of a scientific field can provide a more comprehensive understanding [117].

In this study, cluster analysis was used to evaluate the primary research directions within this field and to highlight the connections between keywords. When keywords frequently co-occur within the same cluster, it signifies that they are linked to the same research domain [6].

In the analysis of all the keywords (*n* = 2995), the term “heavy metals” was the most frequently used, appearing 396 times and accounting for 13.22% of all keywords. It was followed by “risk assessment” (171 occurrences, 5.71%), “cadmium” (160 occurrences, 5.34%), “lead” (137 occurrences, 4.57%), “trace elements” (130 occurrences, 4.34%), and “contamination” (100 occurrences, 3.34%). The remaining keywords had fewer than 100 occurrences each. These observations indicate that assessment of human health risks associated with lead and cadmium contamination in food products is a trending research focus.

Regarding the Author Keywords, out of a total of 1586 keywords, “heavy metals/heavy metal” was the most commonly used author keyword, appearing 204 times (12.86% of the total). It was followed by “risk assessment” (100 occurrences, 6.31%), “food safety” (66 occurrences, 4.16%), and “health risk assessment” (58 occurrences, 3.66%). The remaining keywords had fewer than 50 occurrences.

In terms of Author Keywords, out of a total of 1652 keywords, “heavy metals” was the most frequently used author keyword, appearing 237 times (constituting 14.35% of the total). It was followed by “cadmium” (128 occurrences, 7.75%), “lead” (111 occurrences, 6.72%), and “trace elements” (105 occurrences, 6.36%). The remaining keywords had fewer than 100 occurrences each.

**Figure 8:**
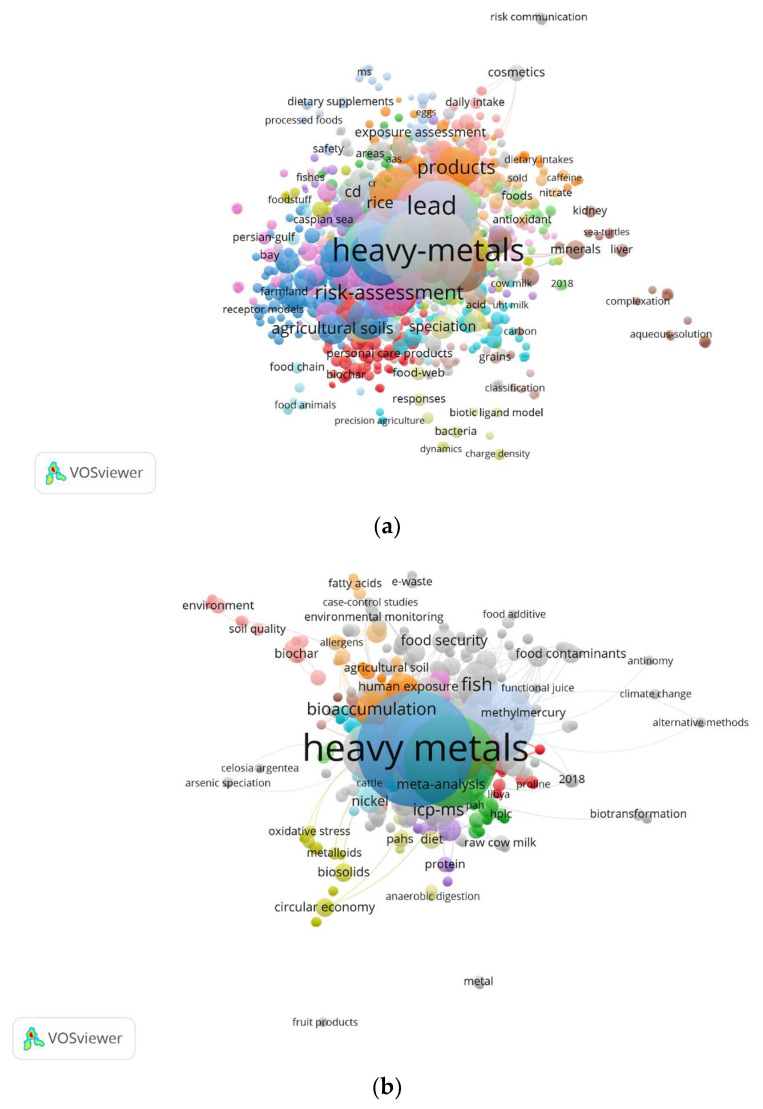

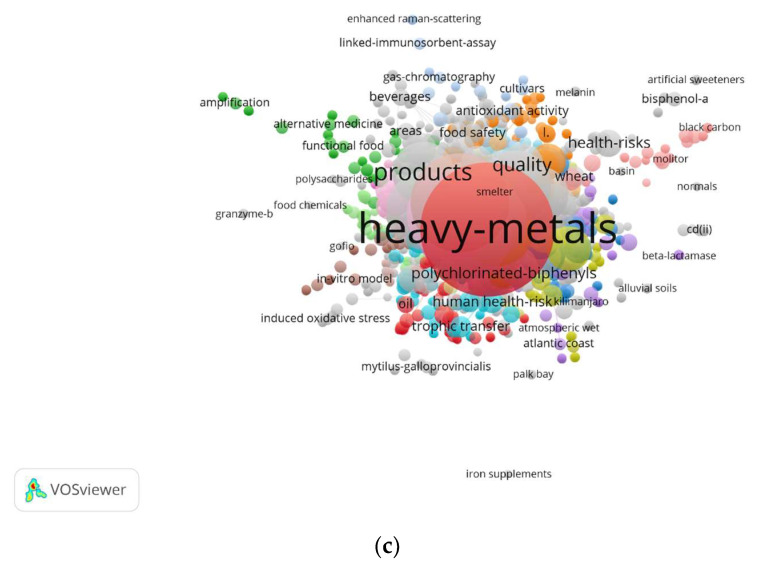
Keyword analysis. (**a**) Co-occurrence network of all keywords. (**b**) Co-occurrence of Author Keywords. (**c**) Co-occurrence of Keywords Plus.

The co-occurrence network of all keywords is presented in Figure 8a, with the keywords categorized into 63 clusters, featuring 41,529 links between these keywords and a strong link strength of 52,720. The most frequent keywords indicating the clusters were “heavy metals” (gray), “cadmium” (gray), “food safety” (green), “risk” (light blue), “lead” (orange), “exposure” (lime), and “contamination” (turquoise). The term “heavy metals” (gray) was located in the core of the network, showing the greatest interest in this topic, which corresponds with the other keywords.

Figure 8b presents the co-occurrence network of author keywords, which were grouped into 80 clusters, 6355 links between these keywords and a strong link strength of 7142. The most frequent keywords indicating the clusters were “heavy metals”, “cadmium”, “risk assessment”, and “lead” (gray), “food safety” and “health risk assessment” (lime). Additionally, the term “heavy metals” (gray) was situated in the core of the network, signifying the greatest interest in this topic, which corresponds with the other keywords.

Figure 8c contains the co-occurrence network of Keywords Plus, which have been organized into 54 clusters. There are 15,519 links connecting these keywords, demonstrating a strong link strength of 20,635. The most prominent keywords that represent these clusters include “heavy metals” (red), “vegetables”, “exposure”, “pollution”, “bioaccumulation”, “trace elements” (gray), “cadmium” (brown), “risk assessment” (lime), and “lead” (pink). Also, the term “heavy metals” (gray) is situated at the core of the network, highlighting its significant relevance in this field, which aligns with other related keywords.

The frequently used keywords primarily relate to metals and their exposure routes, with limited association with diseases or other risk characterizations. Current research on heavy metal health risk primarily focuses on identifying exposure pathways and assessing whether risk levels exceed established thresholds, as determined by quantitative risk indices [4].

## 4. Conclusions and Future Trends

This bibliometric analysis has yielded a total of 573 publications related to the health risk assessment of heavy metals in food products, revealing a consistent increase in publications on this topic, particularly in the last five years. The majority of these publications were scientific articles categorized under the Environmental Science section of the WoS.

In terms of institutional productivity, the Ministry of Agriculture and Rural Affairs, Shahid Beheshti University of Medical Sciences, and the Chinese Academy of Sciences in China emerged as the most productive affiliations. China, despite starting relatively late in terms of publications, has become the leading country in this field, followed by Iran and Italy.

The most frequently cited journals for articles exported from the Web of Science were *Science of the Total Environment*, *Pollution Research*, *Journal of Food Composition and Analysis*, and the *International Journal of Environmental Research and Public Health*.

Among the highly cited studies, Miraglia et al. (2009) received the highest number of citations, followed by Hussain et al. (2017) and Lu et al. (2017). Amin Mousavi Khaneghah from Poland stands out as the most prolific author in this field, with 24 published papers, followed by Yadollah Fakhri from Iran and Nabi Shariatifar, also from Iran. The top five frequently used keywords in these publications were “heavy metals”, “risk assessment”, “cadmium”, “lead”, and “trace elements”.

The articles in the literature review primarily focused on contaminant levels in various food products, with a significant emphasis on fish and seafood, cereals, dairy products, meat and meat products, as well as fruits and vegetables. Some articles highlighted the exceedance of hazard quotient, hazard index, or carcinogenic risk limit values following chronic consumption of the tested samples, indicating the necessity for more stringent control measures to ensure consumer safety.

Current risk assessment is primarily focused on individual substances. However, followed the combined exposure to multiple chemicals within a food product, adverse effects can occur, even if each individual substance in the mixture is below the safety threshold. Therefore, it is essential to perform a collaborative study involving researchers from various fields to conduct a cumulative risk assessment of these “chemical cocktails” present in food products and to assess their impact on both human health and the environment.

Another emerging trend involves use of deterministic Monte Carlo simulation methodology or the ImproRisk model for conducting accurate assessments of chronic dietary exposure concerning the chemical compounds found in the tested matrices.

Furthermore, the adoption of next-generation risk assessment (NGRA) is widely regarded as a future direction. Implementing new scientific insights and innovative approaches in hazard and exposure assessments to effectively meet regulatory requirements has proven challenging [118].

A possible future tool involved in risk assessment is artificial intelligence, which can be integrated into chemical risk assessment, potentially improving understanding of toxicity, reducing animal testing, risk prediction and treatment development [119].

## Figures and Tables

**Table 1:** Data source and search strategy (adapted after Niknejad et al., 2022 [20], Scherbakova and Bredikhin, 2021 [19]).

Description	Results
Documents	573
Period	2000–2022
Sources (journals, books)	573
Authors	3089
Number of papers having co-authors |P+|	558
Number of papers having one author |P1|	15
Authors of single-authored documents	3089
Authors of multi-authored documents (2/3/4/5/6/7/8/9)	246/71/29/17/ 11/10/5/4/
Maximum number of authors in a single paper	9
Organizations	1131
Co-authors per document	5.39
Keywords plus	1652
Countries	95
Author keywords	1586
Total citations	13,224
Average citation per document	23.08
Document type	
Article	475
Review	88
Early access	17
Proceedings	13
Editorial material	4
Book chapter	2

**Table 3:** List of top 10 journals, number of publications and citation on research associated with health risk assessment of heavy metals from food products.

Rank	Name of the Journal	Publications	Citations	Citation Means per Document	H-Index_2022_	IF_2022_	JCR Partition	JIF Quartile
1	*Environmental Science and Pollution Research*	36	715	19.86	154	5.8	Environmental Sciences, 67/274	Q1
2	*Science of the Total Environment*	25	805	32.2	317	9.8	Environmental Sciences, 26/274	Q1
3	*Journal of Food Composition and Analysis*	23	231	10.04	130	4.3	Chemistry Applied, 19/72	Q2
4	*International Journal of Environmental Research and Public Health*	22	497	22.59	167	4.614	Public, Environmental & Occupational Health, 45/182	Q1
5	*Environmental Monitoring and Assessment*	20	288	14.4	132	3	Environmental Sciences, 156/274	Q3
6	*Food Additives and Contaminants, Part A: Chemistry, Analysis, Control, Exposure, and Risk Assessment*	19	560	29.47	57	2.9	Chemistry, Applied, 30/72	Q2
7	*Biological Trace Element Research*	18	155	8.61	94	3.9	Endocrinology & Metabolism, 64/15	Q2
8	*Food and Chemical Toxicology*	15	997	66.47	192	4.3	Toxicology, 20/94	Q1
9	*Food Control*	15	330	22.0	149	6	Food Science & Technology, 24/142	Q1
10	*Environmental Geochemistry and Health*	14	106	7.57	84	4.2	Water Resources, 27/103	Q2

## Data Availability

The study was conducted using articles accesed via Web of Science and then processed using VOSviewer software.
